# Acute gastrointestinal injury in children on ECMO: an analysis of risk factors

**DOI:** 10.3389/fmed.2026.1786101

**Published:** 2026-03-24

**Authors:** Liting Hou, Jiaqi Li, Bin Yan, Weikai Wang

**Affiliations:** 1 Gansu University of Chinese Medicine, Lanzhou, China; 2 Gansu Provincial Maternal and Child Health Hospital, Lanzhou, China

**Keywords:** acute gastrointestinal injury, AGI, children, extracorporeal membrane oxygenation, risk factors

## Abstract

**Objective:**

To investigate the risk factors associated with acute gastrointestinal injury (AGI) during extracorporeal membrane oxygenation (ECMO) therapy in pediatric patients.

**Methods:**

This retrospective study included 62 pediatric patients who were treated with ECMO at Gansu Provincial Maternal and Child Health Hospital and Xi’an Children’s Hospital, Shaanxi Province, from January 2019 to May 2025. Patients were categorized into two groups according to the European Society of Intensive Care Medicine (ESICM) criteria: the significant AGI group (patients with grade II or higher AGI) and the non-significant AGI group (patients without AGI or with grade I AGI). General characteristics and clinical and laboratory parameters were compared between the groups. Binary logistic regression was used to analyze risk factors for significant AGI.

**Results:**

Significant AGI occurred in 28 (45.16%) patients, while non-significant AGI occurred in 34 (54.84%) patients. Univariate analysis revealed that the duration of ECMO, pre-ECMO blood lactate levels, the vasopressor–inotrope score (VIS), the enteral nutrition initiation time, and pre-ECMO proton pump inhibitor (PPI) use significantly differed between the groups (*P* < 0.05). Logistic regression analysis revealed that the ECMO duration, pre-ECMO blood lactate levels, and pre-ECMO PPI use were independent risk factors for significant AGI in pediatric patients receiving ECMO, whereas early (within 48 h) initiation of enteral nutrition was a protective factor.

**Conclusion:**

The incidence of significant AGI in pediatric patients receiving ECMO therapy is high. Elevated pre-ECMO lactate levels, prolonged ECMO duration, and pre-ECMO PPI use are independent risk factors for significant AGI, whereas early enteral nutrition (within 48 h) is a protective factor.

## 1 Introduction

Extracorporeal membrane oxygenation (ECMO), an advanced life support technology for critically ill pediatric patients, is associated with a relatively high complication rate. According to a 2022 report by the Extracorporeal Life Support Organization (ELSO), the overall incidence of ECMO-related complications ranges from 50 to 70% (1). Various individual complications have been reported in the literature, with rates ranging from 5 to 70% (2). Acute gastrointestinal injury (AGI) is a common complication in critically ill patients. Studies indicate that the severity of gastrointestinal injury is strongly correlated with adverse outcomes such as the length of hospital stay, infection risk, and mortality (3). Pediatric gastrointestinal barriers are immature and characterized by high mucosal permeability, unstable microbiota, and weak immune defenses, which increase the susceptibility of children to AGI (4). Few previous studies have specifically addressed risk factors for AGI during pediatric ECMO therapy. Therefore, this study aims to systematically investigate independent risk factors for significant AGI during pediatric ECMO through a two-center retrospective analysis, through which we provide evidence for early clinical identification and intervention.

## 2 Materials and methods

### 2.1 Research subjects

This study, which was approved by the Ethics Committee of Gansu Provincial Maternal and Child Health Hospital (approval number: GSFY Ethics 002), collected data from 98 children who underwent ECMO at Gansu Provincial Maternal and Child Health Hospital and Xi’an Children’s Hospital between January 2019 and May 2025. After the specified criteria were applied, 62 patients were included, while 36 patients were excluded for various reasons, such as a pre-ECMO AGI diagnosis (*n* = 26), ECMO initiation within 24 h (*n* = 4), and incomplete clinical data (*n* = 6). The study included 27 male and 35 female subjects.

### 2.2 Inclusion and exclusion criteria

The inclusion criteria were as follows: ➀ an age ≤ 18 years and ➁ receipt of ECMO after admission. The exclusion criteria were as follows: ➀ reaching the diagnostic criteria for AGI before the initiation of ECMO, ➁ an ECMO start time of < 24 h, and ➂ incomplete collection of clinical data.

### 2.3 AGI diagnostic criteria

This study utilized the definition and classification criteria of acute gastrointestinal injury (AGI) established by the European Society for Critical Care Medicine in 2012 (5). Prior to the initiation of ECMO, none of the children fulfilled the diagnostic criteria for AGI, and all AGI events that occurred during ECMO treatment were new occurrences. This classification system categorizes AGI into four grades: grade I AGI involves transient gastrointestinal dysfunction with a clear and reversible cause, without damage to organ function; grade II AGI involves impaired digestive and absorptive functions of the gastrointestinal tract, leading to failure to meet the body’s nutritional requirements; grade III AGI involves gastrointestinal failure that does not improve following clinical intervention; and grade IV AGI involves gastrointestinal failure accompanied by dysfunction of distant organs, potentially associated with shock and multiple organ dysfunction.

On the basis of these AGI grading criteria, patients were divided into two groups for comparison: the significant AGI group (grade II or higher AGI, *n* = 28) and the non-significant AGI group (no AGI/grade I AGI, *n* = 34). This grouping strategy aims to identify risk factors that lead to significant clinical outcomes on the premise that grade II or higher AGI indicates clear organic dysfunction that is significantly associated with poor prognosis, whereas non-AGI/grade I AGI represents transient, self-limiting functional disturbances. This grouping strategy aligns with that used in previous studies (6, 7).

### 2.4 Observation indicators

1. General patient information, including age, sex, body mass index (BMI), and primary disease status, was collected. 2. AGI diagnostic indicators were gathered. Specifically, new gastrointestinal symptoms following ECMO catheterization were documented through daily bedside records. These symptoms included massive gastric retention (5), diarrhea (defined as watery stools more than three times per day), gastrointestinal bleeding (as evidenced by hematemesis, melena, or bloody gastric juice), altered bowel sounds (either weakened or absent), and feeding intolerance (an inability to achieve 20 kcal/kg/d (5) within 72 h of initiating enteral nutrition). The occurrence and resolution times of all symptoms were recorded with reference to the ECMO initiation date. 3. Clinical data were compiled as follows: ➀ ECMO on-machine indicators, including the ECMO flow rate, mode, systolic blood pressure prior to initiation, diastolic blood pressure, and mean arterial pressure (MAP); and ➁ blood gas analysis and laboratory indicators obtained 24 h before and after ECMO initiation, such as pH, lactate (Lac) levels, partial pressure of oxygen (PO2), partial pressure of carbon dioxide (PCO2), bicarbonate (HCO3-), white blood cell count, red blood cell count, hemoglobin, and albumin levels, were obtained. 4. The following treatment-related indicators were recorded: the maximum vasoactive drug index within 24 h after ECMO initiation (vasopressor-inotrope score, VIS), duration of mechanical ventilation, duration of continuous renal replacement therapy (CRRT), duration of ECMO treatment, length of ICU stay, initiation of enteral nutrition within 48 h, and use of proton pump inhibitors (PPIs) prior to ECMO initiation.

### 2.5 Handling of missing values

In this study, some data for certain laboratory indicators were missing. To mitigate potential bias associated with complete case analysis, multiple interpolation methods were employed. The Markov chain Monte Carlo (MCMC) algorithm was used to generate five complete datasets that were individually analyzed by multivariate logistic regression. Ultimately, the effect values were aggregated and estimated in accordance with the Rubin rule.

### 2.6 Nutritional support and strategies

Enteral nutrition management followed guidelines for critical care nutritional support (8, 9). Early enteral nutrition is defined as the initiation and continuous administration of enteral nutrition via a nasogastric or nasojejunal tube within 48 h of ECMO treatment. Delayed/non-initiated enteral nutrition refers to initiation after 48 h of ECMO treatment or the absence of enteral nutrition throughout the ECMO period.

### 2.7 Statistical methods

Statistical analysis was performed using SPSS version 31.0. Data with a normal distribution are reported as the mean ± standard deviation (χ ± s), and comparisons between groups were conducted using a *t*-test. For data with a skewed distribution, values are presented as medians and quartiles [M (P25, P75)], with the rank sum test employed for group comparisons. Categorical data are expressed as percentages, and theχ^2^ test was used for intergroup comparisons. The Box–Tidwell method was used to assess the linear relationship between continuous variables and logit(P). In the logistic regression model, each continuous variable and its interaction term with the natural logarithm were simultaneously included. If the interaction term was not statistically significant (*P* > 0.05), the linearity hypothesis was deemed valid. All variables with *P* < 0.05 in the univariate analysis were incorporated into the multivariate logistic regression model. The Enter method facilitated variable selection, and the odds ratio (OR) and its 95% confidence interval (CI) were calculated.

## 3 Results

Among the 62 pediatric patients included in the analysis, 28 developed significant AGI, representing an incidence rate of 45.16%. Among this group were 10 male patients and 18 female patients.

### 3.1 Comparison of general data from the two groups

As shown in Table 1, the baseline characteristics of the two groups were comparable, with no significant differences in age, sex, body mass index, or underlying disease (all *P* > 0.05).

**TABLE 1:** Comparison of the general characteristics between two groups [M (P25, P75), $\bar{x}$ ± s].

Indicator	Non-significant AGI group (*n* = 34)	Significant AGI group (*n* = 28)	T/χ ^2^/Z	*P*
Sex (male/female)	17/17	10/18	1.275	0.309
Age (n)			–0.08	0.936
<28 days	4 (11.76)	2 (7.14)		
28 days ∼ < 1 year	4 (11.76)	5 (17.86)		
1 ∼ < 3 years	4 (11.76)	3 (10.71)		
3 ∼ < 6 years	5 (14.71)	3 (10.71)		
6 ∼ < 12 years	11 (32.35)	12 (42.86)		
≥12 years	6 (17.65)	3 (10.71)		
BMI (Z)	0.06(–0.60, 1.50)	–0.025(–1.34, 0.88)	–1.16	0.249
Disease type (n)			3.279	0.525
Fulminant myocarditis	19 (55.88)	12 (42.86)		
ARDS	12 (35.29)	10 (35.71)		
Septic shock	0 (0.00)	1 (3.57)		
Congenital heart disease	3 (8.82)	4 (14.29)		

Age groups are formatted as “lower limit <upper limit,” which indicates that the lower limit is included while the upper limit is excluded. For example, “1∼ < 3 years old” includes patients who were 1 year old but excludes patients who were 3 years old.BMI z-scores were derived from the CDC growth reference.

### 3.2 Results of single-factor and multifactor analyses

The univariate analysis included disease severity indicators, treatment-related parameters, and laboratory metrics. The results indicated that among the analyzed variables, VIS, pre-ECMO lactate levels, ECMO treatment duration, enteral nutrition initiation time, and pre-ECMO PPI use were significantly associated with the occurrence of AGI (all *P* < 0.05). The complete results of the univariate analysis are detailed in Tables 2–4.

**TABLE 2:** Comparison of treatment-related data between two groups [M (P25, P75),x̄ ± s].

Indicator	Non-significant AGI group (*n* = 34)	Significant AGI group (*n* = 28)	T/χ ^2^/Z	*P*
ECMO Initiation mode (VV/VA)	8/26	9/19	0.572	0.57
ECMO duration (h)	158.21 ± 74.48	230.21 ± 96.58	–3.314	0.002
ECMO flow (L/min)	1.44 (0.86, 2.28)	1.4 (1, 2.36)	–0.45	0.66
Systolic blood pressure (mmHg)	90.01 ± 20.95	90.25 ± 28.26	–0.038	0.97
Diastolic blood pressure (mmHg)	57.91 ± 15.04	56.68 ± 16.63	0.305	0.76
Mean arterial pressure (mmHg)	68.33 ± 15.68	67.21 ± 19.18	0.252	0.8
Mechanical ventilation time (h)	229.5 (59.5, 300.75)	241 (147.25, 420.75)	–1.52	0.13
Length of ICU stay (d)	15.5(10, 22.25)	17.5 (12.5, 25.75)	–0.985	0.329
CRRT duration (h)	168 (24, 244)	154 (0, 328.25)	–0.2	0.85
Urine output (mL)	662 (371.5, 1452.5)	781.5 (421.75, 1436.75)	–0.205	0.84
VIS (points)	24.5 (12.5, 41.25)	62.5 (27.75, 79.5)	–2.87	0.004
Time to initiation of enteral nutrition (< 48 h)	30	12	14.469	0.001
PPI use before ECMO (n)	8	19	12.073	0.001

VIS = Dopamine (μg/kg/min) + Dobutamine (μg/kg/min) + 10 × Milrinone (μg/kg/min) + 100 × Epinephrine (μg/kg/min) + 100 × Norepinephrine (μg/kg/min) + 10,000 × Vasopressin (U/kg/min).

**TABLE 3:** Comparison of blood gas parameters before and after mechanical ventilation in both groups [M(P25, P75), x̄ ± s].

Indicator	Non-significant AGI group (*n* = 34)	Significant AGI group (*n* = 28)	T/χ ^2^/Z	*P*
PH				
Pre-	7.37 ± 0.13	7.35 ± 0.07	0.719	0.48
24 h on ECMO	7.4 (7.33, 7.45)	7.43 (7.33, 7.5)	–1.133	0.26
PO_2_ (mmHg)				
Pre-	115.08 (62.75, 181.5)	82.2 (61.25, 154.5)	–0.79	0.44
24 h on ECMO	74.5 (43.75, 119.25)	73.5 (43, 128.25)	–0.042	0.97
PCO_2_ (mmHg)				
Pre-	32.4 (25.03, 46.2)	36.75 (26.28, 43.63)	–0.736	0.47
24 h on ECMO	32.15 (26.58, 38.08)	32.7 (24.53, 40.5)	–0.396	0.7
Na^+^ (mmoL/L)				
Pre-	139.66 ± 4.47	140.96 ± 5.73	–1.004	0.32
24 h on ECMO	141.16 ± 5.25	142.82 ± 6.49	–1.116	0.27
K^+^ (mmoL/L)				
Pre-	3.96 (3.69, 4.5)	3.86 (3.64, 4.5)	–0.226	0.83
24 h on ECMO	3.81 ± 0.59	3.77 ± 0.57	0.267	0.79
HCO_3_^–^(mmoL/L)				
Pre-	19.1 (14.05, 23.85)	20 (14.58, 24.23)	–0.233	0.82
24 h on ECMO	19.37 ± 4.08	21.32 ± 6.24	–1.487	0.14
BE (mmoL/L)				
Pre-	–4.5 (–9.33, –0.28)	–5.65 (–10.45, 0.03)	–0.219	0.83
24 h on ECMO	–4.78 ± 4.33	–2.94 ± 6.72	–1.304	0.2
Lac (mmoL/L)				
Pre-	2.35 (0.9, 3.8)	3.5 (2.33, 6.45)	–2.63	0.008
24 h on ECMO	1.05 (0.68, 1.63)	1.65 (0.55, 2.8)	–1.09	0.28

**TABLE 4:** Comparison of laboratory data between two groups [M(P25,P75),$\bar{x}$ ± s].

Indicator	Non-significant AGI group (*n* = 34)	Significant AGI group (*n* = 28)	T/χ ^2^/Z	*P*
WBC (×10^9^/L)				
Pre-	8.58(5.5, 14.17)	8.43(5.5, 14.36)	–0.233	0.82
24 h on ECMO	10.12(6.23, 15.86)	8.74(5.86, 11.96)	–0.934	0.36
RBC (×10^12^/L)				
Pre-	3.96 ± 0.80	4.06 ± 0.85	–0.471	0.64
24 h on ECMO	3.78(3.37, 4.2)	3.78(3.39, 4.06)	–0.283	0.78
Hb (g/L)				
Pre-	119.04 (103.75, 129.25)	116.5 (96.25, 134)	–0.149	0.89
24 h on ECMO	109.5 (100.75, 122.5)	108.5 (99, 120.25)	–0.58	0.58
Neutrophil count (×10^9^/L)				
Pre-	6.52 (3.32, 10.06)	5.08 (3.34, 10.52)	–0.48	0.64
24 h on ECMO	7.81 (4.7, 13.09)	6.84 (4.51, 9.87)	–0.729	0.47
Neutrophil percentage (%)				
Pre-	71.55 (58.8, 80.55)	73 (63.43, 80.4)	–0.502	0.62
24 h on ECMO	80.75 (68.28, 86.5)	81.6(74.23, 87.05)	–0.431	0.67
Lymphocyte count (×10^9^/L)				
Pre-	1.84 (1.04, 3.72)	1.81 (0.91, 2.8)	0.078	0.94
24 h on ECMO	1.21 (0.79, 1.61)	1.2 (0.75, 1.56)	–0.417	0.68
Lymphocyte percentage (%)				
Pre-	22.6 (12.4, 29.48)	20.7 (12.33, 30.48)	–0.177	0.86
24 h on ECMO	12.4 (9.23, 22.7)	13.25 (9.73, 17.85)	–0.17	0.87
Albumin (g/L)				
Pre-	37.56 ± 6.05	35.35 ± 7.38	1.295	0.2
24 h on ECMO	36.2 (33.56, 37.61)	37.67 (34.37, 42)	–1.747	0.08
Total bilirubin (μmoL/L)				
Pre-	17.05 (9.9, 33.61)	13.15 (9.7, 19.7)	–1.125	0.264
24 h on ECMO	67.799 (36.38, 81.38)	69.36 (33.3, 74.93)	–0.191	0.852
Indirect bilirubin (μmoL/L)				
Pre-	10.8 (5.55, 21.5)	6.95 (5.33, 15.21)	–1.386	0.168
24 h on ECMO	45.80 (17.93, 51.67)	43.57 (24.68, 53.39)	–0.014	0.991
Direct bilirubin (μmoL/L)				
Pre-	4.2 (0.3, 8.7)	3.64 (1.83, 9.18)	–0.036	0.975
24 h on ECMO	12.81 (7.78, 16.25)	12.9 (10.79, 17.16)	–0.729	0.471
ALT (U/L)				
Pre-	31.5 (17.75, 68.25)	55.5 (20.75, 176.35)	–1.584	0.114
24 h on ECMO	272.72 (36.75, 333.41)	308.05 (50, 353.54)	–1.117	0.267
AST (U/L)				
Pre-	102.5 (38.6, 237.5)	113.25 (54.88, 419.5)	–1.096	0.277
24 h on ECMO	622.54 (101.55, 680.96)	467.15 (87.03, 654.78)	–0.566	0.576

The results of the Box–Tidwell test reveal that all the continuous variables are linearly related to logit(P), satisfying the linearity assumption of logistic regression. Multivariate logistic regression analysis revealed elevated lactate levels prior to ECMO initiation (OR = 1.445; 95% CI: 1.011–2.064; *P* = 0.043).

A prolonged ECMO duration (OR = 1.013; 95% CI: 1.003–1.023; *P* = 0.008) and pre-ECMO PPI use (OR = 7.367; 95% CI: 1.529–35.485; *P* = 0.013) were independent risk factors for significant AGI, whereas the initiation of enteral nutrition within 48 h (OR = 0.2; 95% CI: 0.04–0.994; *P* = 0.049) was a protective factor, as shown in Table 5.

**TABLE 5:** Multivariate analysis of factors associated with gastrointestinal dysfunction during ECMO.

Risk factor	OR	95% CI	*P*
ECMO duration (h)	1.013	1.003–1.023	0.008
VIS (points)	1.018	0.992–1.045	0.175
Lac (mmoL/L)	1.445	1.011–2.064	0.043
Time to enteral nutrition (< 48 h)	0.2	0.040–0.994	0.049
PPI use before ECMO initiation	7.367	1.529–35.485	0.013

### 3.3 Clinical outcomes

Among the 62 children in the study cohort who underwent ECMO, 46 (74.2%) survived and were subsequently discharged from the hospital, while 16 (25.8%) did not survive. The mortality rate in the significant AGI group was 39.2% (11/28), whereas that in the non-significant AGI group was 14.7% (5/34). This difference was statistically significant (*P* < 0.05). However, there was no statistically significant difference in the length of ICU stay, as illustrated in Table 6.

**TABLE 6:** Comparison of clinical outcome data between the two groups [M (P25, P75), x̄ ± s].

Indicator	Non-significant AGI group (*n* = 34)	Significant AGI group (*n* = 28)	T/χ ^2^/Z	*P*
Length of ICU stay (d)	15.5 (10.0, 22.3)	17.5 (12.5, 25.8)	–0.985	0.329
Case fatality rate (n%)	5 (14.7)	11 (39.2)	4.845	0.041

### 3.4 Early enteral nutrition and clinical outcomes

Among the 62 children included in this study, 42 were assigned to the early enteral nutrition group (initiated within 48 h), while 20 were assigned to the non-early enteral nutrition group. The mortality rate in the early enteral nutrition group was 16.7%, which was significantly lower than the 45.0% observed in the non-early enteral nutrition group, with this difference reaching statistical significance (*P* < 0.05). No statistically significant difference was found in the duration of ECMO or the length of ICU stay between the two groups (Table 7).

**TABLE 7:** Comparison of early enteral nutrition and clinical outcome-related data [M (P25, P75), x̄ ± s].

Indicator	Early enteral nutrition group(<48 h)	Non-early enteral nutrition group	T/χ ^2^/Z	*P*
ECMO duration (h)	181.29 ± 90.6	210.55 ± 93.67	1.177	0.244
Length of ICU stay (d)	16 (10, 25)	17 (11, 23)	–0.06	0.955
Case fatality rate (n%)	7 (16.7)	9 (45)	5.581	0.029

### 3.5 Comparison of gastrointestinal symptom spectra between the two groups

This study examined the gastrointestinal symptoms in two groups of children undergoing ECMO. The incidences of abdominal distension, vomiting/gastric retention, and gastrointestinal bleeding were significantly higher in the significant AGI group compared to the non-significant AGI group (*P* < 0.05). Diarrhea, intestinal perforation, and constipation were infrequent in both groups (*P* > 0.05). Feeding intolerance was noted in 8.8% (3/34) of the non-significant AGI group. In the significant AGI group, only 12 cases initiated enteral feeding, of which 5 cases experienced intolerance, representing 41.7% of the initiators, suggesting a potential selection bias. Consequently, direct comparison between the two groups may not be appropriate. No organic injuries, including gastrointestinal bleeding, were identified in the non-significant AGI group, which adhered to the AGI classification criteria. The low incidence of diarrhea is likely attributable to stringent definitions and the early parenteral nutrition strategy employed during ECMO (Table 8).

**TABLE 8:** Comparison of gastrointestinal symptom spectra between the two groups.

Symptoms	Non-significant AGI group (*n* = 34)	Significant AGI group (*n* = 28)	Test value	*P*
Abdominal distension	4 (11.8%)	11 (39.3%)	6.341	0.017
Vomiting/gastric retention	7 (20.6%)	16 (57.1%)	8.793	0.004
Diarrhea	2 (5.9%)	1 (3.6%)		1^1^
Gastrointestinal hemorrhage	0	17 (60.7%)	28.441	<0.001
Diminished bowel sounds	0	3 (10.7%)		0.087^1^
Intestinal perforation	0	1 (3.6%)		0.452^1^
Constipation	0	1 (3.6%)		0.452^1^

^1^Fisher’s exact probability test was employed; χ^2^-tests were used for the remainder. Due to the limitations inherent in retrospective study designs, this research was unable to ascertain the precise timeframe for improvement in each symptom.

### 3.6 Comparison of baseline clinical characteristics between the early enteral nutrition group and the non-early enteral nutrition group of children

Among the 62 children included in this study, 42 were assigned to the early enteral nutrition group (initiated within 48 h), while 20 were placed in the non-early enteral nutrition group. The VIS score and lactic acid levels in the early enteral nutrition group were significantly lower than those in the non-early enteral nutrition group (*P* < 0.05). However, no statistically significant difference was observed in the duration of ECMO between the two groups (*P* > 0.05). The aforementioned baseline differences were adjusted for in the multivariate analysis, as detailed in Table 9.

**TABLE 9:** Comparison of baseline clinical characteristics between the early enteral nutrition group and the non-early enteral nutrition group of children.

Indicator	Early enteral nutrition group(<48 h)	Non-early enteral nutrition group	T/χ ^2^/Z	*P*
ECMO duration (h)	181.29 ± 90.6	210.55 ± 93.67	1.177	0.244
VIS	28.5 (15.25, 48)	53 (24.75, 83.75)	–2.35	0.018
Lac	2.6 (1.4, 3.85)	3.72 (2.25, 7.28)	–2.215	0.026

**FIGURE 1:**
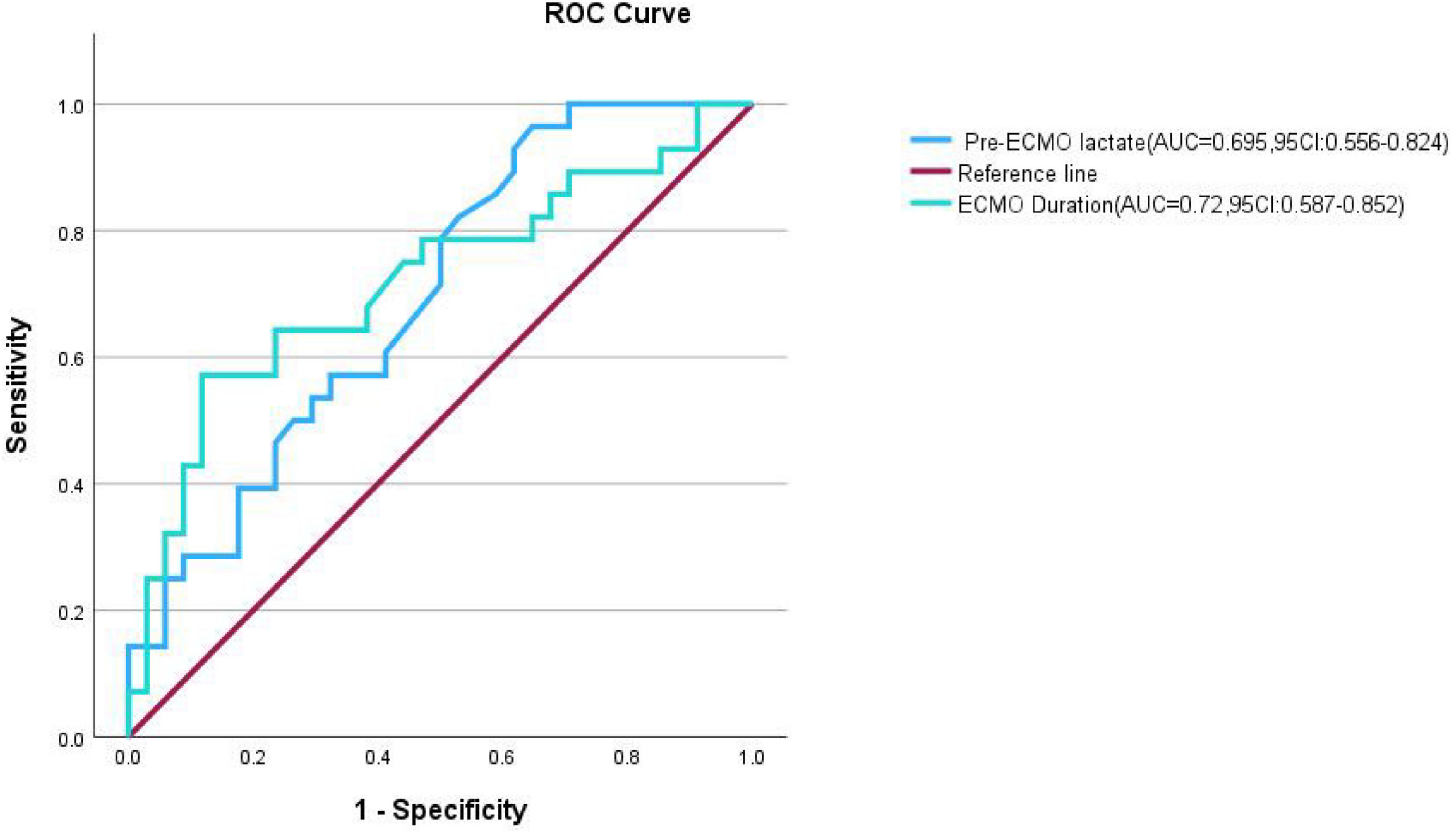
Receiver operating characteristic curves of pre-ECMO lactic acid levels and ECMO duration.

Multivariate analysis of meaningful quantitative data yielded receiver operating characteristic curves. Pre-ECMO lactate levels and the duration of ECMO support were significantly different (*p* < 0.05). The calculated critical values were a lactate concentration of 1.45 mmol/L and an ECMO duration of 229.5 h (Figure 1).

## 4 Discussion

This study demonstrates that prolonged ECMO duration, elevated lactate levels prior to ECMO initiation, and preinitiation use of PPIs are independent risk factors for significant AGI in children. The ESICM consensus states that all critically ill pediatric patients are at high risk for AGI. Factors such as systemic hypoperfusion (10), cytokine storm (5, 11), sedation and analgesia, and medications can all impair gastrointestinal function, with an incidence rate as high as 60% (12). The non-pulsatile blood flow associated with ECMO exacerbates tissue hypoperfusion (13), which further increases the risk and severity of gastrointestinal injury and thereby impacts prognosis. Therefore, the identification of risk factors for clinically significant AGI during ECMO therapy in pediatric patients and the implementation of early interventions are critical.

This study demonstrated that the vasoactive drug index (VIS) within 24 h was significantly associated with the occurrence of acute gastrointestinal injury (AGI) and may be linked to intestinal hypoperfusion induced by high-dose vasoactive drugs (14). However, the VIS was not identified as an independent predictor in the multivariate analysis. Pearson correlation analysis revealed a weak correlation between the auxiliary time of extracorporeal membrane oxygenation (ECMO) and the VIS score (*r* = 0.261; *P* = 0.041), while no significant correlation was found with lactate levels (*r* = -0.005; *P* = 0.968). Additionally, the VIS score was weakly correlated with lactate levels (*r* = 0.256; *P* = 0.044). The variance inflation factor (VIF) values for each variable (lactate, 1.077; ECMO treatment duration, 1.079; VIS score, 1.155) suggest that multicollinearity was not a serious concern within the model. As a quantitative indicator of circulatory failure (15), the VIS was strongly correlated with lactate levels, which reflect tissue perfusion, and the duration of ECMO treatment, which indicates disease severity. When these two factors were included in the multifactorial model, the independent effect of VIS was diminished. Consequently, while VIS can serve as a clinical indicator for AGI risk, addressing tissue hypoperfusion and managing primary disease are more fundamentally important for preventing AGI occurrence.

After adjusting for confounding factors, multivariate logistic regression revealed several independent risk and protective factors. Elevated lactate levels indicate severe hypoxia and inadequate perfusion (16), and the resulting visceral ischemia directly impairs intestinal barrier function. Furthermore, lactate acts as a danger signal that promotes the acetylation of high mobility group box 1 (HMGB1), which exacerbates inflammation and may trigger multiple organ dysfunction (17). A prolonged ECMO duration exacerbates intestinal injury through various mechanisms. In addition to the microcirculatory impairment caused by secondary infection and sepsis (9, 18), the systemic inflammatory response induced by ECMO itself disrupts tight junction proteins and accelerates epithelial apoptosis (11). Simultaneously, non-pulsatile blood flow impairs mesenteric vascular regulation, which causes occult visceral ischemia, whereas deep sedation suppresses gastrointestinal motility. Therefore, clinicians must adhere to established guidelines (9), and ECMO should be discontinued as early as possible when withdrawal criteria are met to reduce the risk of significant AGI. Previous studies have demonstrated that early initiation of enteral nutrition during pediatric ECMO therapy is associated with reduced mortality (19). Multivariate analysis revealed that early enteral nutrition was an independent protective factor against significant AGI (OR = 0.2, corresponding to an 80% risk reduction).

Multivariate analysis indicated that early enteral nutrition serves as an independent protective factor against significant acute gastrointestinal injury (OR = 0.2, 80% risk reduction). Additionally, mortality rates were markedly lower in the early enteral nutrition group compared to the non-early enteral nutrition group, suggesting that its protective effect may enhance survival by alleviating gastrointestinal injury and decreasing infection risks. Although no significant differences were noted between the two groups regarding ECMO duration or ICU length of stay—factors potentially influenced by cardiopulmonary recovery and other variables—early enteral nutrition maintains its clinical significance. However, it should be noted that EEN implementation is influenced by the severity of the pediatric patient’s condition: Univariate analysis revealed that children in the EEN group had lower VIS scores and lactate levels than those in the non-EEN group (*P* < 0.05), suggesting clinicians tend to initiate early feeding in less critically ill patients. This phenomenon aligns with clinical practice logic but carries the risk of confounding by indication. Evidence-based guidelines (20) indicate that early enteral nutrition improves intestinal blood flow, promotes intestinal motility, reduces bacterial translocation, and accelerates mucosal repair. Although reports on gastrointestinal complications associated with enteral nutrition vary across studies—for instance, Hanekamp et al. (21) research suggested it may increase risks of feeding intolerance such as abdominal distension and gastric residual volume, while Pérez et al. (22) study found no significant increase in gastrointestinal complication risk—both studies recommend early initiation of enteral nutrition. Consequently, this study concludes that implementing early enteral nutrition during ECMO therapy offers both physiological advantages and clinical outcome benefits. It is recommended that clinical practice should individualize implementation based on the specific condition of the pediatric patient. For those with stable hemodynamics, low-dose enteral nutrition should be initiated as early as possible.

The increased use of proton pump inhibitors (PPIs) in clinical practice has led to various complications. The potent inhibition of gastric acid secretion by PPIs can induce structural changes in the gastric mucosa, thereby impacting nutrient absorption (23). Research has demonstrated that PPIs can modify the composition of the intestinal flora, increasing the risk of bacterial intestinal infections from pathogens such as *Clostridium difficile*, Salmonella, and Campylobacter (24). A meta-analysis (25) revealed that PPIs contribute to gastrointestinal dysfunction by promoting bacterial overgrowth in the small intestine, compromising the mucosal barrier, and stimulating the release of bacterial products. When assessing the relationship between PPI use and significant acute gastrointestinal injury (AGI), it is essential to consider potential confounding biases. Children with more severe disease are already strong candidates for prophylactic PPI use and also face a markedly increased risk of AGI because of underlying pathological conditions such as hemodynamic instability and inadequate perfusion. Consequently, the association identified in this study is more likely attributable to the confounding factor of disease severity rather than a direct pharmacological effect of PPI use. The current data do not sufficiently support an independent causal relationship between PPI use and significant AGI. Although this study adjusted for several known confounding factors using multivariate logistic regression, the inherent limitations of retrospective observational studies hindered our ability to entirely exclude residual confounding arising from unmeasured or inadequately measured variables. The study had a limited sample size of 62, with 28 significant AGI events identified. After 5 independent variables were incorporated into the multivariate logistic regression, the events per variable (EPV) was approximately 5.6, which is below the conventional EPV ≥ 10 threshold suggested by Peduzzi et al. (26). Despite van Smeden et al.’s (27) criticism of the insufficient evidence supporting this criterion, a low EPV could result in model overfitting and unreliable parameter estimation. Consequently, caution is advised when interpreting the study findings, underscoring the need for validation in larger prospective studies in the future. Moreover, while the binary classification of AGI used in the study is straightforward for clinical purposes, this classification method may not fully capture the continuous progression of disease. Although this classification method is supported by the literature and is commonly employed in critical care research, combining grade I AGI patients with entirely healthy individuals could obscure the distinction between critical conditions and subclinical disease or organ dysfunction. The combining of these patients might underestimate the impact of certain risk factors in the early stages of AGI. Finally, diagnosing AGI relies on clinical parameters such as abdominal symptoms and gastric retention volume, introducing subjective judgments that could introduce measurement bias.

## 5 Conclusion

In conclusion, preliminary research suggests that elevated lactate levels prior to the initiation of ECMO and prolonged ECMO treatment duration are linked to a significant occurrence of AGI, whereas early enteral nutrition may serve as a protective factor. This conclusion offers clinical evidence for the early identification of AGI. Timely identification and intervention can improve the prognosis for children. Although a significant association between the use of PPIs and AGI was noted in the multivariate analysis, it is essential to remain vigilant against confounding bias. These findings require further validation through multi-center, large-sample prospective studies.

## Data Availability

The datasets generated and/or analyzed during the current study are not publicly available due to patient privacy and ethical considerations but are available from the corresponding author on reasonable request.
